# Centenarian

**Published:** 1915-10

**Authors:** 


**Centenarian.** The Ind. Med. Jour. mentions the funeral, July 10, of Bartholomew Finn at Muncie. He was 106 years old.

